# Interaction between illness cognitions and dyadic coping: a qualitative exploration of stress adaptation in young and middle-aged colorectal cancer patients and their spouses

**DOI:** 10.1007/s00520-026-10463-x

**Published:** 2026-03-08

**Authors:** Qian Sun, Peirong Xu, Yuee Wen, Lei Ruan, Xuelan Liu, Junsheng Peng, Janelle Yorke, Ka Yan Ho

**Affiliations:** 1 School of Nursing, Sun Yat-Sen University, Guangzhou, Guangdong China; 2 School of Nursing, The Hong Kong Polytechnic University, 11 Yuk Choi Road, Hung Hom, Kowloon, Hong Kong SAR, China; 3 Gastrointestinal Surgery Unit, Sixth Affiliated Hospital, Sun Yat-Sen University, Tianhe District, 26 Erheng Road, Guangzhou, Guangdong China; 4 Department of Colorectal Surgery, The Sixth Affiliated Hospital of Sun Yat-Sen University, Guangzhou, Guangdong China; 5 Guangdong Provincial Key Laboratory of Colorectal and Pelvic Floor Diseases, Guangdong Institute of Gastroenterology, Supported By National Key Clinical Discipline, Guangzhou, Guangdong China

**Keywords:** Colorectal cancer, Illness cognitions, Dyadic coping, Stress adaptation, Qualitative research

## Abstract

**Purpose:**

To explore the stress adaptation experiences of young and middle-aged couples with colorectal cancer (CRC), specifically examining the interaction between illness cognitions and dyadic coping.

**Methods:**

Using purposive sampling, semi-structured interviews were conducted with eight pairs of young and middle-aged CRC couples, along with eight patients and five spouses, at a tertiary hospital in Guangzhou from October 2023 to February 2024. Data were analyzed following the six-stage process outlined in the interpretative phenomenological analysis research guidelines, with coding and organization supported by Nvivo 12.0 software to extract hierarchical themes reflecting the interaction process between illness cognitions and dyadic coping in CRC couples.

**Results:**

Three themes emerged: (1) Intrapersonal dynamics: positive illness cognitions facilitated adaptive coping strategies, whereas negative cognitions triggered maladaptive coping behaviors. (2) Dyadic mechanisms: a cross-partner influence was observed where one partner’s illness cognitions affected the other’s coping through specific pathways, including negative resonance, reverse activation, and compensatory adaptive coping. (3) Key moderators: relationship intimacy, communication quality, family resilience, social support, family role identity, and division of labor significantly moderated these interactions.

**Conclusions:**

The findings reveal complex bidirectional influences between CRC couples, including compensatory and reverse activation mechanisms. Relationship intimacy, communication quality, family role identity, resilience, and social support play crucial moderating roles in facilitating or hindering adaptive coping. These results underscore the necessity of psychosocial interventions adopting a family systems perspective, focusing on enhancing communication skills, clarifying role division, and strengthening support networks to improve psychological adjustment in cancer-affected families.

## Introduction

According to the World Health Organization, over 1.92 million new colorectal cancer (CRC) cases occurred worldwide in 2022, making it the third most common malignancy, with about 900,000 deaths, ranking as the second in cancer-related mortality [1]. By 2030, CRC incidence and mortality are projected to rise by 60%, reaching 2.2 million new cases and 1.1 million deaths [2]. Both rates are increasing, with onset shifting to younger ages, creating a substantial and escalating disease burden [3, 4]. While survival rates have improved, stress adaptation—particularly among young and middle-aged patients—remains underexplored despite its crucial role in quality of life and long-term outcomes [5, 6]. In this study, “young and middle-aged adults” were defined as individuals aged 18 to 59 years [7]. Cancer patients within this age range face distinct challenges—such as career disruption, parenting demands, and shifting dyadic dynamics—that significantly complicate their cancer coping processes [8, 9].

Cancer is a profound multidimensional stressor, producing significant psychological impacts on patients and spouses. Psychological distress in CRC patients typically escalates in the first year post-diagnosis before stabilizing, reflecting cancer’s role as an acute stressor that triggers existential anxiety and mortality concerns, necessitating adaptive coping strategies [10, 11]. The impact extends across the couple system, with evidence of clinically significant distress in both partners at diagnosis and substantial declines in mental health and sexual well-being during the first year [12]. Spousal caregivers face higher physical and emotional burdens than other caregiver groups, increasing their vulnerability to depression and distress [13].

The systemic transactional model (STM) views illness as a shared “We-Disease,” where cognition and coping mutually influence each other in dyadic relationships [14]. Enhancing stress communication and cognitive restructuring can improve couples’ adaptive capacity and psychological well-being [14]. Illness cognition—the understanding, evaluation, and interpretation of one’s health, including helplessness, acceptance, and perceived benefits—and dyadic coping, the joint strategies partners use to manage stress, significantly affect psychological distress in young and middle-aged CRC couples [14–16]. Family resilience, social support, and communication quality shape dyadic coping [17–19], while effective, supportive, and problem-solving communication fosters adaptation, and avoidance or conflict undermines it [20, 21]. Despite growing literature, gaps remain in understanding how illness cognitions and dyadic coping interact over the stress adaptation process. This study addresses these gaps by focusing on young and middle-aged CRC patients and their spouses, examining the reciprocal influences of illness cognitions and dyadic coping within the STM framework, and identifying factors shaping these processes. Findings aim to inform targeted interventions to enhance psychosocial support and strengthen adaptive capacity in this population.

## Methods

### Study design

This qualitative study, grounded in a phenomenological perspective, explored how CRC patients and spouses experience and adapt to stress, focusing on the interaction between illness cognitions and dyadic coping. Specifically, we used interpretative phenomenological analysis (IPA), which does not only focus on individuals’ descriptions of phenomena, but also emphasizes an in-depth interpretation of the underlying meanings of their experiences [22, 23]. Given its suitability for complex emotional dynamics in dyadic contexts, IPA was selected. The study followed the Comprehensive Standard Guide for Qualitative Research Reports [24] and was approved by the Ethics Committee of the School of Nursing, Sun Yat-sen University (No. L2023SYSU-HL-004).

### Setting and sampling

The study was conducted from October 2023 to February 2024 in the Department of Gastrointestinal Surgery and the Department of Medical Oncology at a tertiary hospital in Guangzhou.

Purposive sampling was used to deliberately select participants who could provide rich, relevant insights for our research questions [25]. Specifically, we recruited young and middle-aged CRC patients meeting the inclusion/exclusion criteria (Table 1). The sample size was determined by code saturation—no new themes or codes emerged after interviews with 16 patients and 13 spouses [26]. Two doctoral students (S.Q. and X.PR.) contacted potential participants, explained the study’s aims and procedures, and invited participation. All contacted individuals agreed to participate in the interviews.

**Table 1:** Eligibility criteria of participants

Eligibility criteria	People with colorectal cancer	Spouses
Inclusion criteria	a) Aged between 20 and 59 years	a) Aged 20 years or older and lived with the patient
	b) Diagnosed with colorectal cancer by histopathology	b) Aware of the patient’s medical condition
	c) Married	c) Served as the patient’s primary caregiver
	d) Able to speak Mandarin and express clearly	d) Able to speak Mandarin
	e) Able to provide informed consent	e) Able to provide informed consent
Exclusion criteria	a) Unaware of their diagnosis of colorectal cancer	a) Had a history of, or currently suffers from, disorders which could affect their consciousness or psychiatric illnesses
	b) Presence of other serious diseases, such as stroke or heart failure	b) Had other serious diseases, such as malignancies, stroke, or heart failure
	c) Individuals with disorders which could affect their consciousness, or psychiatric illnesses	c) Currently receiving psychological treatment or participating in similar trials
	d) Concurrent malignancies at other sites or recurrence of tumors	
	e) Currently receiving psychological treatment or participating in similar trials	

### Data collection

This study employed semi-structured interviews guided by protocols developed by a research team with expertise in oncology care (Table 2). Semi-structured interview is a qualitative research method in which the researcher develops an interview guide based on the study’s questions and objectives prior to the interview, using it as a framework or prompt during the conversation. In practice, the researcher maintains flexibility, allowing the discussion to flow naturally rather than rigidly adhering to the guide’s sequence [27]. Currently, qualitative interviews involving family members are primarily conducted in three formats: individual interviews, dyadic interviews, and a combination of both [28]. Individual interviews enable participants to speak openly from a personal viewpoint, free from concern about their partner’s reactions, making them suitable for sensitive topics. Dyadic interviews involve patients and spouses together, eliciting shared narratives and revealing relational dynamics. Combined approaches integrate both perspectives, offering a more comprehensive understanding [29].

**Table 2:** Interview outlines

Patients with colorectal cancer	Spouses
1. When were you diagnosed, and what symptoms did you experience?	1. How did you feel when you first learned about your partner’s diagnosis, and what impact did the illness have on both your life and your partner’s life?
2. How did you feel when you first learned about your diagnosis, and what impact has the illness had on your life and your spouse’s life?	2. How did your partner react at that time, and in what ways did their attitude affect you?
3. How did your spouse react, and in what ways has their attitude affected you?	3. How do you and your partner discuss issues related to cancer?
4. How do you and your spouse discuss issues related to cancer?	4. In what ways has your relationship changed as a result of the illness, and what do you think caused these changes?
5. In what ways has your relationship changed as a result of the illness, and what do you think caused these changes?	5. Throughout the process of coping with the disease, what kind of support has your partner provided to you, and how would you evaluate that support?
6. Throughout the process of coping with the disease, what kind of support has your spouse provided, and how would you evaluate that support?	

To thoroughly explore how illness cognitions and dyadic coping interact in young and middle-aged CRC couples, we adopted a combined format. Both complete dyads and eligible individuals (when one partner declined or was ineligible) were included as interviewees. After obtaining participants’ informed consent, two doctoral students (S.Q. and X.P.R.) conducted face-to-face interviews in a distraction-free conversation room, with S.Q. serving as the primary interviewer and X.P.R. providing assistance and maintaining detailed records. Each interview session lasted a minimum of 30 min.

### Data analysis

This study utilized NVivo 12.0 to systematically organize and analyze qualitative data. NVivo efficiently manages diverse data types—including text and audio—enabling rapid categorization and extraction of key information. Following the six-stage process outlined in the IPA research guidelines [30], each transcript was read multiple times for immersion, with detailed initial noting to capture descriptive, linguistic, and conceptual features. Experiential statements were then formulated, and emergent themes were developed and clustered. Cross-case analysis was conducted to identify shared and unique patterns among participants, culminating in the construction of an interpretative narrative supported by participant quotations. 

### Rigor

To enhance the rigor of this qualitative study, we focused on credibility, confirmability, and transferability. Credibility was ensured by inviting participants to review and verify the themes and subthemes identified during analysis and by involving two independent experts in oncology nursing to cross-check and validate the coding. Any inconsistencies were addressed through in-depth team discussions until consensus was reached. We recruited participants from multiple departments to capture a broad spectrum of experiences. To ensure the fidelity of translation from Chinese to English, a systematic translation and back-translation procedure was employed. Confirmability was supported by maintaining transparency throughout the research process, including regular team meetings to examine assumptions and analytic decisions. All researchers received specialized training in qualitative methods to ensure methodological rigor. For transferability, we provided comprehensive details on the study context, participant recruitment, data collection and analysis, and participant characteristics, enabling readers to assess the relevance and applicability of the findings to other contexts.

## Results

In this study, we interviewed eight patient-spouse pairs jointly and eight patients and five spouses individually. Among the patients, ten were male and six were female, aged 33 to 59 years (mean age: 45.19). Twelve held a bachelor’s degree, 11 were diagnosed with rectal cancer, and 9 were at stage IV. All had adenocarcinoma, with time since diagnosis ranging from 15 days to 30 months. Detailed patient characteristics are provided in Table 3. Of the spouses, four were male and nine were female; eight held a bachelor’s degree. Ten were employed full-time, with caregiving durations from 11 days to 16 months and marriage length ranging from 8 to 35 years. Further details are presented in Table 4.

**Table 3:** The demographics of colorectal cancer patients (*N* = 16)

ID	Gender	Age (years)	Education level	Occupation	Disease diagnosis	Time since diagnosis	Disease stage	Pathological type	Interview method
P1	Male	33	University	Full-time	Colon cancer	1 month	Stage IV	Adenocarcinoma	Joint interview
P2	Female	59	University	Full-time	Rectal cancer	2 months	Stage II	Adenocarcinoma	Joint interview
P3	Male	59	High school	Retired	Rectal cancer	4 months	Stage III	Adenocarcinoma	Joint interview
P4	Female	49	University	Unemployed	Rectal cancer	4 months	Stage IV	Adenocarcinoma	Joint interview
P5	Male	48	Middle school	Self-employed	Rectal cancer	8 months	Stage IV	Adenocarcinoma	Joint interview
P6	Male	53	University	Full-time	Rectal cancer	11 months	Stage III	Adenocarcinoma	Joint interview
P7	Male	41	University	Full-time	Colon cancer	7 months	Stage IV	Adenocarcinoma	Joint interview
P8	Female	39	University	Full-time	Rectal cancer	16 months	Stage III	Adenocarcinoma	Joint interview
P9	Male	42	University	Full-time	Colon cancer	4 months	Stage IV	Adenocarcinoma	Individual interview
P10	Male	46	University	Self-employed	Rectal cancer	9 months	Stage IV	Adenocarcinoma	Individual interview
P11	Female	41	University	Full-time	Rectal cancer	11 months	Stage IV	Adenocarcinoma	Individual interview
P12	Male	35	University	Full-time	Rectal cancer	13 months	Stage IV	Adenocarcinoma	Individual interview
P13	Female	38	University	Self-employed	Rectal cancer	24 months	Stage IV	Adenocarcinoma	Individual interview
P14	Male	41	University	Full-time	Rectal cancer	4 months	Stage III	Adenocarcinoma	Individual interview
P15	Female	55	High school	Retired	Colon cancer	30 months	Stage II	Adenocarcinoma	Individual interview
P16	Male	44	High school	Self-employed	Colon cancer	15 days	Stage III	Adenocarcinoma	Individual interview

**Table 4:** The demographics of spouses (*N* = 13)

ID	Gender	Age (years)	Education level	Occupation	Cumulative caregiving duration	Living with the patient	Years of marriage	Interview method
S1	Female	35	University	Full-time	1 month	Yes	8	Joint interview
S2	Male	60	University	Full-time	2 months	Yes	35	Joint interview
S3	Female	57	High school	Retired	4 months	Yes	34	Joint interview
S4	Male	49	University	Full-time	4 months	Yes	25	Joint interview
S5	Female	48	Middle school	Full-time	8 months	Yes	22	Joint interview
S6	Female	50	University	Full-time homemaker	11 months	Yes	28	Joint interview
S7	Female	39	University	Full-time	7 months	Yes	9	Joint interview
S8	Male	41	University	Full-time	16 months	Yes	15	Joint interview
S9	Female	40	Middle school	Full-time	6 months	Yes	20	Individual interview
S10	Female	39	Middle school	Self-employed	8 months	Yes	14	Individual interview
S11	Male	53	University	Full-time	11 days	Yes	32	Individual interview
S12	Female	50	High school	Full-time	10 months	Yes	24	Individual interview
S13	Female	47	University	Full-time	4 months	Yes	20	Individual interview

### Thematic analysis

**Fig. 1:**
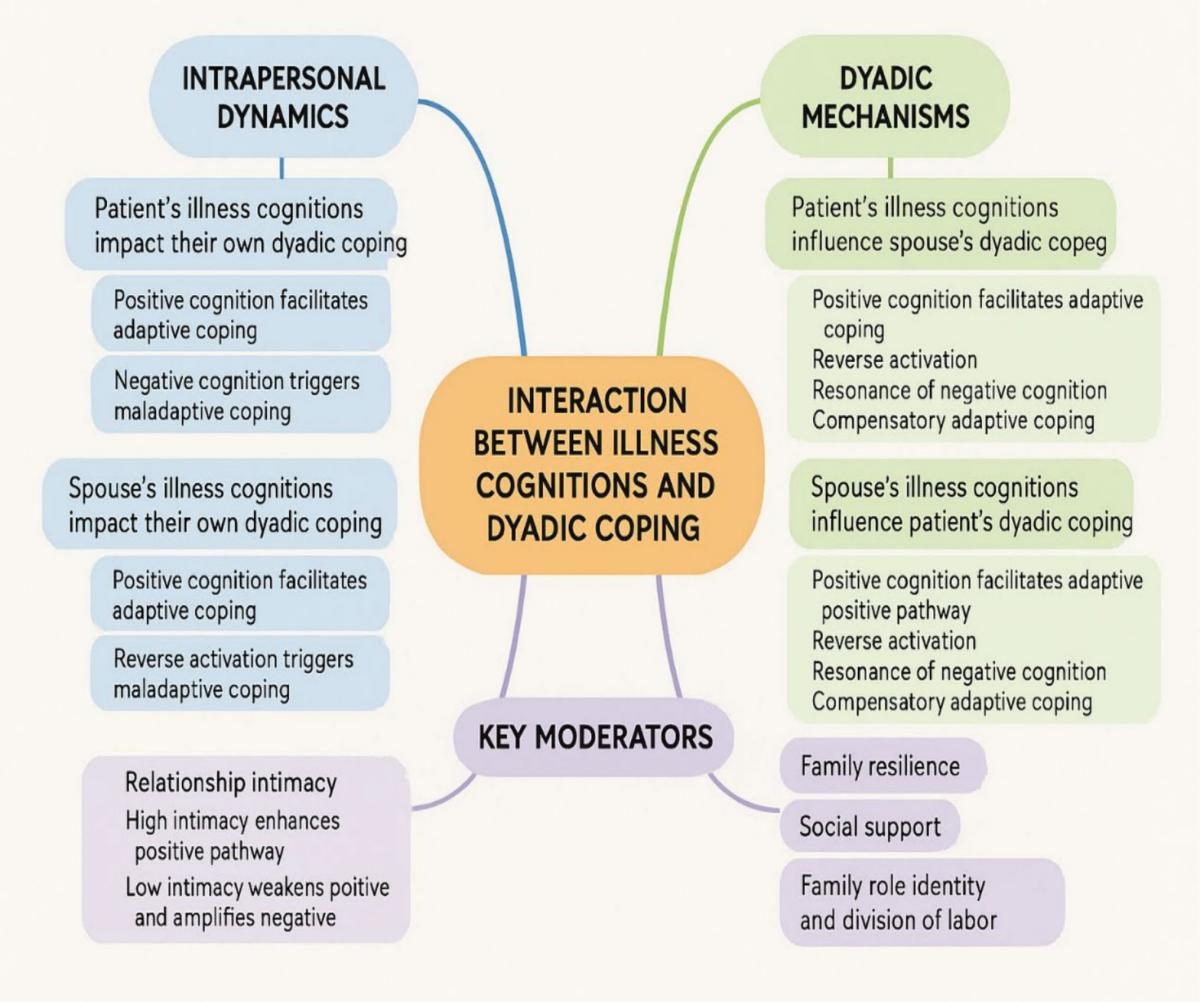
A framework of themes and subthemes

By analyzing the experiences of stress adaptation in both patients and their spouses from a dyadic perspective, we identified three themes which described the interaction processes of illness cognitions and dyadic coping, including intrapersonal dynamics between illness cognitions and dyadic coping, dyadic mechanisms of illness cognitions and dyadic coping, and key moderators in the interaction process (Fig. 1).

### Intrapersonal dynamics between illness cognitions and dyadic coping

#### Subtheme 1: The impact of patients’ illness cognitions on their own dyadic coping behaviors

1. Positive illness cognitions facilitate adaptive dyadic coping
  Many patients reported that, after the initial shock of diagnosis, they gradually developed acceptance and hope, sometimes finding new meanings in life. This positive outlook enabled them to approach treatment more rationally, seek support, communicate openly with family, and adapt their lifestyles—demonstrating strong adaptability and agency.
  I quickly accepted the illness. I don’t think it’s a big deal, getting sick is just a part of the life. I’ll focus on treatment, no matter what the illness is. I trust the hospital and I trust science. These days, most serious illnesses can be treated. If it really can’t be cured, then nothing more can be done, but I know the doctors will do their best. (P3, Male, 59 years, Rectal cancer, Stage III)
  After getting sick, I’ve gained more insight into life. I cherish life and the present moment much more, can figure out a lot of things, and face them positively. I would discuss with my family where to have surgery and make treatment decisions together. (P11, Female, 41 years, Rectal cancer, Stage IV)
2. Negative illness cognitions trigger maladaptive dyadic coping
  Some patients, especially early in their diagnosis or during treatment, experienced intense helplessness and hopelessness. They described “collapsing,” losing confidence, and even wanting to give up. In this state, they often withdrew emotionally, avoided communication, and resisted support—typical of maladaptive coping.
  When I first heard about my diagnosis, I almost had a breakdown. I just couldn’t believe this was happening to me. I don’t drink, I rarely eat barbecue, and my diet is only mildly spicy. The only habit I have is smoking, but I always thought my lifestyle was pretty healthy. When I first found out (that I had cancer), I had no idea what to do. (P6, Male, 53 years, Rectal cancer, Stage III)
  During chemotherapy, I have experienced numbness and stiffness all over my body, and I couldn’t speak. The whole process was miserable. At that point, I thought, if I die, so be it—I just didn’t want to continue treatment or cooperate with the doctors, and I also avoided communicating with my family. (P2, Female, 59 years, Rectal cancer, Stage II)

#### Subtheme 2: The impact of spousal illness cognitions on their own dyadic coping behaviors

1. Positive illness cognitions facilitate adaptive dyadic coping
  When spouses adopted positive illness cognitions—such as acceptance and perceived benefits—they were more likely to engage in adaptive coping, including proactive care, encouragement, taking on more family responsibilities, and seeking external resources. This mindset boosted their self-efficacy, strengthened emotional bonds, and promoted collaborative coping, providing patients with increased support and security.
  My husband often encourages me, saying that as long as treatment is possible, we should pursue it and do our best. We’re on the same page. We want to tackle the illness actively, and if it can be cured, that’s wonderful. (P2, Female, 59 years, Rectal cancer, Stage II)
2. Negative illness cognitions trigger maladaptive dyadic coping
  When spouses experience negative illness cognition—such as helplessness—they might display maladaptive dyadic coping behaviors, including emotional coldness, criticism, reduced communication, and family disengagement.
  When he (the patient) is in a bad mood, he takes it out on me, which of course makes me feel bad as well. Whenever he loses his temper, the atmosphere at home becomes extremely tense. The children become very cautious when they see him angry, even breathing is difficult. I don’t even want to talk to him about it (the diagnosis and treatment of CRC). (S10, Female, 39 years, married for 14 years)
  When we first found out about the illness, I was truly devastated. I couldn’t face him and felt really down. There were several times when I went outside to cry, but I never dared to cry in front of him because I didn’t want to affect his mood. We communicate less and less, and both feel lonely. (S6, Female, 50 years, married for 28 years)

### Dyadic mechanisms of illness cognitions and dyadic coping

#### Subtheme 1: The influence of patients’ illness cognitions on spousal dyadic coping

1. Patient’s positive illness cognitions facilitate spousal adaptive dyadic coping
  In some families, when patients displayed optimism and proactive acceptance regarding their illness, their spouses were often positively influenced by this attitude, demonstrating greater emotional resonance, support, and collaboration.
  My spouse accepted the reality of his illness and faced it courageously. This, in turn, encouraged me. Since he was facing it positively, I definitely wanted to accompany him and face it together. (S12, Female, 50 years, married for 24 years)
2. “Reverse activation”: patient’s positive illness cognitions and spousal maladaptive dyadic coping
  In some families, a mismatch between illness cognitions and coping emerged. Even when patients were able to positively accept cancer and actively cooperate with treatment, their spouses could not adjust their own cognition and emotions, displaying emotional indifference, avoiding communication, and reducing caregiving, which were forms of maladaptive dyadic coping.
  I feel that I have already accepted this illness and try my best to cooperate with the doctor’s arrangements, but my husband seems completely unconcerned about my situation. He hardly takes care of things at home, let alone accompanies me to the hospital. Many times, I go to chemotherapy alone, and when I come back, he rarely asks about it. (P13, Female, 38 years, Rectal cancer, Stage Ⅳ)
3. Resonance of patient’s negative illness cognitions and spousal maladaptive dyadic coping
  When patients experienced ongoing helplessness or despair, spouses were often similarly affected, exhibiting anxiety, withdrawal, reduced communication, or emotional distancing. This mutual reinforcement of negative emotions could intensify a stifling home environment, weaken the family’s support system, and create a vicious cycle of negative illness cognitions and maladaptive coping.
  After his stoma surgery, he was in a bad mood and lost his temper with me every day. Naturally, I also felt down and didn’t want to discuss (the stoma) with him anymore. I just let him be. (S10, Female, 39 years, married for 14 years)
4. Patient’s negative illness cognition and spousal compensatory adaptive copingPatient’s negative illness cognition and spousal compensatory adaptive coping
  In some families, despite the patient’s despair, the spouse responded by taking on additional caregiving responsibilities, seeking information, and providing emotional and practical support. This compensatory coping helped maintain family functioning and emotional stability, offering the patient vital external and psychological resources.
  He couldn’t accept having cancer. During chemotherapy, no companions were allowed, so he was alone for treatment, couldn’t eat, lost his temper, and said he didn’t want treatment anymore. I kept comforting and encouraging him, telling him not to give up. Afterwards, I never left him alone again—I accompanied him every time. After all, what’s happened has happened, and we have to face it. (S6, Female, 50 years, married for 28 years)

#### Subtheme 2: The influence of spousal illness cognitions on patients’ dyadic coping

1. Spousal positive illness cognitions promote patients’ adaptive dyadic coping
  When spouses demonstrated optimism and a proactive attitude toward illness, patients were often positively influenced. They became more willing to cooperate with treatment, express emotions, participate in decision-making, and adapt their lives—exhibiting adaptive dyadic coping. These dynamics enhanced patients’ self-efficacy and strengthened family cohesion.
  My husband is always very optimistic and keeps encouraging me, saying, ‘Don’t worry, we’ll definitely get through this.’ He goes with me to the hospital every day and helps me look up different treatment options. Seeing how confident he is, I feel I can’t give up, and I’m more willing to cooperate with the doctors and focus on treatment. (P2, Female, 59 years, Rectal cancer, Stage II)
  My wife always says, ‘Let’s face this together, no matter how hard it is.’ She often plans meals and exercise routines with me. The more positive she is, the more motivated I feel, and my mood has improved a lot compared to before. (P14, Male, 41 years, Rectal cancer, Stage III)
2. “Reverse activation”: spousal positive illness cognitions and patients’ maladaptive dyadic coping
  In some families, even when spouses were highly optimistic and proactive, patients might feel pressured to “stay strong,” experience unmet emotional needs, or diminished self-worth. As a result, they might withdraw, avoid communication, or become passive—manifesting maladaptive coping. This “reverse activation” underscored that excessive positivity from a spouse could sometimes increase the patient’s psychological burden.
  My wife always says, ‘Don’t worry, everything will be fine,’ and keeps encouraging me to stay positive and cooperate with treatment. But when I was going through chemotherapy, it was really tough—I wasn’t as strong as she imagined, and I didn’t really want to share my true feelings with her because I was afraid of disappointing her. (P12, Male, 35 years, Rectal cancer, Stage Ⅳ)
  He’s always researching, scheduling follow-ups, and encouraging me to exercise more. Honestly, sometimes I really don’t want to deal with it—the more positive he is, the more I want to avoid it. (P15, Female, 55 years, Colon cancer, Stage Ⅱ)
3. Resonance of spousal negative illness cognitions and patients’ maladaptive dyadic coping
  When spouses remained in a state of pessimism, anxiety, or helplessness, patients were also prone to negative emotions, manifesting as emotional withdrawal, avoidance of communication, reduced adherence, self-blame, or passive resignation. This resonance of negative emotions and behaviors could intensify a stifling family atmosphere, weaken support, and create a vicious cycle of negative illness cognitions and maladaptive coping.
  He (the spouse) is still pretty worried… I often see him looking down, maybe struggling to accept what’s happened. He seems even more stressed than I am. (P4, Female, 49 years, Rectal cancer, Stage IV)
  He (the spouse) always thinks I won’t get better, doesn’t arrange anything, and doesn’t encourage me. Sometimes I feel so lonely that I don’t even want to keep up with treatment. (P8, Female, 39 years, Rectal cancer, Stage Ⅲ)
  She (the spouse) is not positive at all now, always complaining about how exhausting it is to take care of me. Hearing that makes me feel terrible, like I’m a burden, so I don’t want to trouble her anymore. (P9, Male, 42 years, Colon cancer, Stage IV)
4. Compensatory mechanism: spousal negative illness cognitions and patients’ adaptive dyadic coping
  In some families, even when the spouses were in a negative cognitive state, the patients actively chose to hide their own pain, take on more family responsibilities, and strive to maintain emotional stability and a positive atmosphere at home. This “compensatory coping” mechanism helped buffer the spouses’ negative emotions and enhanced the family’s adaptability.
  My spouse has been really pessimistic lately, always worried that I won’t get better and sometimes crying in secret. Seeing her so upset, I feel I have to be stronger, so I comfort her and tell her we’ll get through this together. (P10, Male, 46 years, Rectal cancer, Stage IV)

### Key moderators in the interaction between illness cognitions and dyadic coping

#### Subtheme 1: The moderating role of relationship intimacy

1. High relationship intimacy: enhancing the positive pathway from cognition to coping
  When intimacy between the patient and spouse was high, the facilitative effect of positive illness cognitions on adaptive dyadic coping became more pronounced. High intimacy provided a secure emotional foundation for both partners, making patients more willing to express their inner feelings and seek support, while enabling spouses to sensitively perceive and actively respond to the patient’s needs. In such contexts, positive cognition was more readily translated into open communication, joint decision-making, and collaborative caregiving—resulting in a virtuous cycle of adaptive dyadic interaction.
  Since my diagnosis, my wife has shown even more care for me. She has devoted more time and energy to both my physical care and emotional support, and as a result, our relationship has grown much closer. Now we can share our thoughts, worries, and plans for the future, and face this together. (P16, Male, 44 years, Colon cancer, Stage III)
2. Low relationship intimacy: weakening the positive pathway and amplifying the negative pathway
  When intimacy was low, positive illness cognitions might not effectively lead to adaptive dyadic coping. Communication barriers, lack of trust, and emotional distance could hinder support and might cause positive intentions to be misunderstood or ignored. Low intimacy also amplified the effects of negative cognitions, increasing the risk of misunderstanding, blame, emotional withdrawal, and other negative interactions.
  We often argue about food, and it happens quite a lot. I try to stop him from eating certain things, but he’s always thinking about what he wants to eat, especially spicy foods. Even I add chili, he says it’s not spicy at all. He might not feel it’s spicy, but his body really can’t handle it. He just thinks I’m trying to control him and our relationship has worsened. So now, I just don’t want to interfere anymore. (S11, Male, 53 years old, married for 32 years)

#### Subtheme 2: The moderating role of communication quality

Communication quality significantly shaped how illness cognitions translated into dyadic coping among young and middle-aged CRC patients and their spouses. High-quality communication fostered the effective transformation of positive illness cognitions into adaptive coping, thereby strengthening family resilience. Conversely, communication barriers could weaken the expression of positive cognitions or amplify the impact of negative cognitions, resulting in maladaptive coping.

1. Open communication: strengthening the positive transformation from illness cognitions to dyadic coping
  In an environment of open and honest communication, patients and spouses could freely share information about the illness, emotional experiences, and practical needs. This promoted mutual understanding, joint decision-making, and timely correction of cognitive biases, enabling positive cognitions to be translated into adaptive coping and enhancing family cohesion and resilience.
  We talk about it and don’t avoid the topic. I even joined an anti-cancer group to see how others are doing and to learn more. When I first found out about the (CRC) diagnosis, I told my wife right away. (P3, Male, 59 years, Rectal cancer, Stage III)
  We never avoid discussing this issue. My wife always tells me everything she knows, and we work together to figure out where to have surgery. We also discuss together what the best treatment is. Even when the medical staff talk about the illness, we explore and make decisions together. (P16, Male, 44 years, Colon cancer, Stage III)
2. Avoidant communication: weakening the positive pathway and amplifying the negative pathway
  In avoidant communication, patients and spouses tended to shy away from illness-related discussions or fail to respond to each other’s emotional needs. This hindered the expression and consensus of positive illness cognitions, making it easier for negative cognitions to manifest as avoidance, indifference, or other maladaptive coping, thereby increasing psychological burden and emotional distance within the family.
  Why talk about (the illness)? (laughs) Talking about it just puts us in a bad mood. There’s no need to discuss it—whatever will be, will be. If we bring it up, it only makes us feel worse, so we try to avoid the topic as much as possible. (S5, Female, 48 years, married for 22 years)
  We rarely talk about it; this illness is our shared pain. In daily life, we just talk about school or the kids. (S13, Female, 47 years, married for 20 years)
3. Passive-absorptive communication: disrupting interactive feedback between illness cognitions and dyadic coping
  Passive-absorptive communication occurred when patients or spouses chose not to express their inner feelings and shouldered emotional burdens alone to avoid troubling the other. This pattern disrupted the feedback loop between cognition and coping, leading to internalized stress. As a result, positive cognitions were less likely to manifest as adaptive coping behaviors, while feelings of loneliness and helplessness might intensify.
  I never talk about my stress; I just find something to learn or do on my own. There’s no point in telling him. (S3, Female, 57 years, married for 34 years)
  Of course, it affects me, but no matter how bad I feel, I will never show it to him. I try to manage my emotions better than he does. I don’t want to make him feel worse. Even when I’m struggling, I may end up comforting him instead. (S10, Female, 39 years, married for 14 years)
4. Conflict communication: distorting the cognition–coping pathway and triggering negative interactions
  Conflict communication was manifested when patients and spouses, due to differences in opinions or excessive stress, engaged in arguments, blame, or emotional distancing during illness management. This mode of communication did not only weaken the effects of positive illness cognition but can also amplify negative illness cognition into hostility, blame, passivity, and other maladaptive coping, creating a vicious cycle within the family system.
  At first, we had a lot of conflicts and arguments because neither of us had any experience with the illness or stoma care. We were both flustered and overwhelmed, and our moods were all over the place. (P14, Male, 41 years, Rectal cancer, Stage III)
  I put my career on hold to take care of you, left our child with the grandparents, and the child was acting up all the time, making the house chaotic. I was by your side every day. Yet, you still lost your temper with me. Besides, it’s not like I caused your illness, so I was feeling pretty upset myself. (S10, Female, 39 years, married for 14 years)

#### Subtheme 3: The moderating role of family resilience

Family resilience, as a dynamic construct, moderated how illness cognitions were transformed into different coping behaviors under the stress of cancer. Highly resilient families were found to be better at leading positive illness cognitions into adaptive coping strategies, but when these highly resilient families confronted negative cognitions, they could mobilize collective resources—such as joint problem-solving, emotional support, and shared stress management—to cope. This collective approach buffered the negative impact of crises and strengthened the family’s overall adaptability.

When I was first diagnosed, the atmosphere at home was very tense, but our whole family would hold meetings to discuss how to arrange treatment and daily life. Although everyone was scared, we encouraged each other and felt that as long as we were together, we could get through anything. (S12, Female, 50 years, married for 24 years)

I think our family’s greatest strength is that we don’t blame each other when problems arise; instead, we work together to find solutions. Whenever I feel down, my family members will proactively talk to me and help share my burdens, which gives me a lot of strength. (P6, Male, 53 years, Rectal cancer, Stage III)

#### Subtheme 4: The moderating role of social support

A high level of social support could greatly enhance patients’ and family members’ sense of security and belonging, reduce anxiety and helplessness, and promote the development of positive illness cognitions. This supportive environment facilitated the transformation of positive illness cognitions into adaptive coping strategies, such as active participation in treatment, effective emotional regulation, and collaborative family functioning. Conversely, a lack of social support could intensify negative cognitions, hinder adaptive coping, and exacerbate family stress and isolation.

My family and friends are all very supportive. After I became ill, they took turns visiting me, helping with cooking, and looking after the children. With them around, I feel that I am not fighting this alone, and I am more confident about cooperating with the doctors. (P4, Female, 49 years, Rectal cancer, Stage Ⅳ)

Our family is quite traditional. My family members didn’t want me to talk much about my illness, fearing it would affect the family atmosphere. At first, I didn’t dare say much to them, but later, after more communication with other patients in support groups, I gradually learned to face the illness directly. (P8, Female, 39 years, Rectal cancer, Stage III)

### Subtheme 5: The moderating role of family role identity and division of labor

Clear family roles and division of labor helped patients accept their limitations with less self-blame, while spouses who took more responsibilities often felt valued. The clarity of family roles supported open communication, mutual support, and effective collaboration, fostering adaptive coping. In contrast, role confusion or imbalance could lead to helplessness in patients, overwhelmed feelings in spouses, poor communication, and increased maladaptive coping.

My wife used to be very confident, but after she got sick, she always felt that others were talking behind her back. She feels inferior now. (S8, Male, 41 years, married for 15 years)

I used to manage everything at home—work, chores, and the children’s schooling. Now, I can’t handle many things anymore, and my spouse has to take care of most of it. (P2, Female, 59 years, Rectal cancer, Stage II)

I leave for work at 7 a.m. and get back after 7 p.m., then I still have to cook, take care of his emotions, and the pressure is really high. We’re both worried about what the future holds for his health. (S9, Female, 40 years, married for 20 years)

## Discussion

This qualitative study provides an in-depth exploration of the interaction between illness cognitions and dyadic coping among young and middle-aged CRC patients and their spouses. Our results confirmed that illness cognitions are not a static individual construct; rather, they are continuously shaped and changed within the dyadic relationship. Positive cognitions (e.g., acceptance, perceived benefits) promote adaptive dyadic coping—open communication, mutual support, and collaborative problem-solving—whereas negative cognitions (e.g., helplessness, hopelessness) precipitate maladaptive responses, including avoidance, emotional withdrawal, and family disengagement. This pattern is consistent with previous research and highlights the importance of illness cognitions for dyadic coping behaviors [31]. Previous research has demonstrated that positive illness cognitions help reduce psychological distress, promote active treatment engagement, and enhance resilience and quality of life [32]. Furthermore, a significant bidirectional influence between illness cognitions and dyadic coping was observed among cancer patients and their spouses in existing literature [15]. Our qualitative results added to the literature by revealing mechanisms such as “reverse activation,” in which one partner’s positive illness cognition did not elicit a synchronous adaptive response from the other, but led to emotional withdrawal or passivity, and “compensatory coping,” that is, one partner’s helplessness could also prompt the other to assume greater emotional or practical responsibilities.

Several key moderating factors are known to shape the interaction between illness cognitions and dyadic coping among young and middle-aged CRC patients and their spouses. These factors are relationship intimacy, communication quality, family resilience, social support, family role identity, and division of labor. Our qualitative findings, adding to extant literature, elucidated how these factors facilitated or hindered the transformation of illness cognitions into adaptive or maladaptive dyadic coping patterns. In this study, relationship intimacy was found to be a secure emotional foundation that enhanced illness cognitions to be transformed into adaptive dyadic coping. Particularly, high intimacy was reported as a crucial factor that fostered openness, emotional resonance, and mutual responsiveness, enabling patients to express inner feelings and seek support, while spouses sensitively perceived and addressed the patients’ needs. The findings align with the study of Leo et al. [33], which found that higher marital intimacy was associated with lower levels of negative affective expression and more effective coping strategies in cancer couples. Conversely, low intimacy weakened the positive influence of illness cognitions and amplified negative pathways, leading to emotional distancing and communication breakdowns [33].

Our study showed that open and honest communication helped transform positive illness cognitions into effective coping strategies like collaborative problem-solving and emotional support, strengthening family cohesion and resilience. In contrast, avoidant, passive, or conflict communication disrupted positive transformation, leading to emotional withdrawal, misunderstandings, and relational strain. These findings align with previous research showing that open communication improves relationship closeness, marital satisfaction, and psychological adjustment in couples facing cancer, while concealment and avoidance harm these outcomes [34, 35]. Our qualitative approach highlighted communication quality as a dynamic mechanism that could either support or hinder stress adaptation. This underscores the importance of interventions focused on enhancing open, supportive communication to improve both individual well-being and family functioning, tailored to the couple’s relational and cultural context.

According to family resilience theory, when high resilience is present within couples experiencing cancer, they are more likely to adapt positively to stress by utilizing both intra- and extra-family resources and employing adaptive coping strategies, which supports their quality of life [36]. Our findings are in line with this theory and illustrate that highly resilient families were more likely to translate positive illness cognitions into adaptive coping strategies. When confronted with negative cognitions, these families could mobilize collective resources—such as joint problem-solving, emotional support, and shared stress management—to buffer the impact of stress. A previous study also showed a positive correlation between family resilience and dyadic coping in lung cancer patients and their spousal caregivers [18]. Therefore, it is imperative to develop intervention programs for cancer-affected families to improve the interactive dynamics between patients and their spouses, explore their positive belief systems, and support them in building resilience.

Social support refers to an individual’s perception of the availability of external assistance [37]. Our findings indicated that higher levels of social support enhanced patients’ and family members’ sense of security and belonging, reduced anxiety and helplessness, and promoted the development of positive illness cognitions. Conversely, insufficient social support reinforced negative cognitions, hindered adaptive coping, and exacerbated family stress and isolation. These results are consistent with a previous study, which reported that higher levels of social support could alleviate depression and anxiety symptoms in breast cancer patients by facilitating functional coping strategies [38]. This qualitative study revealed multiple moderating factors which formed a complex and interconnected network that shaped how illness cognitions evolved into dyadic coping responses. The interplay between relationship intimacy and communication quality was found to be very crucial in our participants, as these two constructs jointly fostered emotional openness and mutual support. Meanwhile, family role identity and division of labor provided structural clarity for families, alongside family resilience and social support offered emotional and practical resources essential for sustained adaptation.

In this qualitative study, clear family role identity and equitable division of labor were found to help patients accept changes in their abilities while fostering a sense of accomplishment and value in their spouses. Also, the clearness in family role identity and division of labor supported open communication and mutual support, promoting adaptive coping. Conversely, role confusion or imbalance exacerbated feelings of helplessness and emotional exhaustion, leading to maladaptive coping and relational distress. Previous researches have shown that in the context of family function changes, the absence of role renegotiation and task reallocation makes patients more prone to helplessness and diminished self-worth, while their partners are at increased risk of emotional exhaustion, leading to avoidance, conflict, and greater psychological distress [39, 40]. These findings highlight the importance of dynamically adjusting family roles and communication to enhance adaptive capacity and reduce conflict.

The findings of this study offer several important implications for clinical practice. Firstly, interventions should address both individual and cross-partner processes by reshaping illness cognitions in both patients and spouses—for example, on one hand, reducing feelings of helplessness, promoting acceptance, and encouraging benefit-finding, while on the other hand, addressing reciprocal influences to disrupt negative resonance and foster compensatory adaptive coping. Secondly, given the mediating role of spousal relationship, enhancing intimacy and communication quality can strengthen positive cognition and buffer against the effects of negative appraisals. Lastly, reinforcing family-level resources is essential for sustaining positive changes. Particularly, building family resilience and social support, together with clarifying role identity and division of labor, can prevent overloading the caregivers, stabilize adaptive dyadic coping, and support long-term psychosocial adjustment. While this study provides rich and contextually grounded insights, some limitations must be acknowledged. First, the sample was restricted to young and middle-aged CRC patients and their spouses in China, which may limit the generalizability of these findings to other populations or cultural contexts. Second, a selection bias regarding socioeconomic status was observed, as the majority of participants possessed a university-level education. This demographic profile might restrict the applicability of our findings to couples with lower educational attainment, who might experience different barriers in health literacy and communication resources. Future research should therefore include more diverse samples across various cultural and socioeconomic backgrounds.

## Conclusion

The findings demonstrated that patients and spouses influenced each other in complex, bidirectional ways, with mechanisms such as compensation and reverse activation shaping their coping processes. Key factors—including relationship intimacy, communication quality, family role identity, resilience, and social support—significantly moderated whether couples achieved adaptive coping. These results highlight the importance of psychosocial interventions grounded in a family-centered approach, emphasizing improved communication, clear role allocation, and robust support networks to enhance psychological adjustment in families facing cancer.

## Data Availability

No datasets were generated or analyzed during the current study.
